# Chief complaints of patients with cancer who visit the emergency department over their oncologist’s outpatient clinic in South Korea

**DOI:** 10.1186/s12904-022-00946-z

**Published:** 2022-04-21

**Authors:** Min Hyun Son, Han Sol Chung

**Affiliations:** Department of Emergency Medicine, College of Medicine, Yeungnam University, 170 Hyeonchung-ro, Nam-gu, Daegu, 42415 Republic of Korea

**Keywords:** Patients with cancer, Cancer pain, Emergency department, Oncologist

## Abstract

**Background:**

There have been several reports of patients with cancer visiting the Emergency Department (ED) rather than the outpatient department of their oncologist. In this study, we aimed to analyze the chief complaints, visit time, and time spent in the ED between study groups of patients with cancer visiting the ED. This finding will help medical staff provide better care for patients with cancer and reduce time spent in the ED.

**Methods:**

A total of 787 patients with cancer visited the Regional Emergency Medical Center between January 1, 2020, and December 31, 2020. After the exclusion criterion such as patients who were transferred to the ED with a referral issued slip, patients who were pregnant women or minors under the age of 18 were applied, data from 607 patients with cancer were collected retrospectively from electronic medical records at the hospital. The participating patients with cancer were divided into two groups: 1) ED group—those who were cared for by the ED physician and 2) Referral group—those who were referred to their oncologist for hospitalization.

**Results:**

We found that 40% of the total patients with cancer included in the study visited the ED with a chief complaint of pain. It was observed that the highest frequency of visits to the ED was around noon during clinic hours. The length of ED stay was 169 and 566 min for the ED and referral groups, respectively.

**Conclusion:**

It would be more beneficial for patients with cancer visiting the ED to be quickly discharged from the ED physician’s active care for their symptoms. This usage of ED services will reduce unnecessary waiting time.

## Background

The incidence of cancer is increasing worldwide. The GLOBOCAN 2020 estimates indicate that there were 19.3 million new cases of cancer and almost 10 million deaths from cancer in 2020 [1]. As a result, the number of patients with cancer visiting the emergency department (ED) is also increasing. A study by Rivera et al. [2] reported that a total of 29.5 million patients with cancer visited the ED in USA alone between 2006 and 2012. Owing to the nature of their illness, patients with cancer usually have an oncologist on call, who is primarily responsible for providing treatment and continuity of care. Nevertheless, a study by Mayer et al. [3] reported a high frequency of patients with cancer visiting the ED rather than the outpatient clinics of their oncologist, even during the working hours of the oncologist. Several attempts have been made to analyze the reasons behind patients with cancer visiting the ED in such cases. For instance, patients with cancer have a high level of fear. They want to see a doctor as soon as possible as they tend to worry that even minor pain is a sign of aggravation or recurrence of cancer [4, 5]. Such psychological insecurity is a cause of frequent ED visits by patients with cancer [6]. Another important cause for this is the difficulty in making outpatient appointments. Moreover, in some countries with large territories, patients with cancer often choose to visit the nearby local ED instead of the hospital where their oncologist is, to avoid long-distance travel [3].

Thus, in this study, we aimed to explore the chief complaints, visit time, and time spent in the ED between study groups of patients with cancer visiting the ED. This finding will help medical staff provide better care for patients with cancer and reduce time spent in the ED.

## Methods

### Study design and population

A total of 787 patients with cancer visited the Regional Emergency Medical Center at Yeungnam University Hospital, which oversees Daegu Metropolitan City and nearby provinces, between January 1, 2020, and December 31, 2020. It is a general ED. We recruited 787 patients with cancer, with existing cancer diagnosis codes, while those incidentally diagnosed for the first time in the ED were excluded. Among them, 171 patients who were referred to the ED for emergency care due to serious conditions while receiving outpatient treatment from their oncologist and those who were transferred to the ED with a referral slip issued by a first- or second-tier hospital were excluded. This is because they did not choose to visit the ED voluntarily and, thus, did not meet the purpose of this study. After excluding nine patients who were pregnant women or minors under the age of 18, we included a total of 607 patients in the study.

### Data collection

We collected patient data retrospectively from electronic medical records (EMR) at the hospital. Data, including patient’s ED diagnosis codes according to the International Classification of Diseases (ICD) 10, age, sex, chief complaint, day and time of visit, proportion of patients referred to the oncologist, length of ED stay (until discharge), emergency severity index (ESI) level, initial vital signs, and the laboratory findings on arrival.

As per the ICD-10 criteria, patients with cancer were identified as patients with C-code.

Moreover, chief complaints were also extracted from the EMR. When a patient visits the Regional Emergency Medical Center at Yeungnam University, the ED physician examines the patient and records a chief complaint in addition to the doctor’s record. This is not a free-form process; rather, the physician selects the most suitable alternative from a pre-programmed list of chief complaint words (abdominal pain, chest pain, fever, shortness of breath, nausea, weakness, etc.). This standardized method was implemented to mitigate the confusion in EMR systems arising from the difference in arbitrary expressions among doctors. The chief complaint data were analyzed by categorizing complaints as per organ system, pain, fever, and others. Although pain and fever are not organ systems, they were categorized independently owing to the multiplicity of the complaints and clear symptoms that cannot be attributed to a specific organ system.

The ESI is a five-level triage algorithm. It was designed to evaluate both patient acuity and anticipated resource utilization necessary to reach disposition. An ESI level one assignment represents the most acute, high-resource utilization patients, while an ESI level five assignment represents patients who are the least acute and resource intense.

### Study group and consultation process

All patients with cancer, brought to the ED, were cared for by the ED physician, and those requiring hospitalization were referred to their oncologist. The former group of patients was defined as the “ED group” and the latter as the “Referral group”.

Referral is done in a way that an ED physician consults their oncologist for hospitalization. Although the ED physician determined the need for hospitalization, the referral was provided if the patient strongly desired to consult their oncologist, despite the lack of medical necessity for admission to a tertiary hospital. However, in this case, the process after referral is an unnecessary wait for hospitalization rather than a treatment process.

Patients without an oncologist were referred to the most appropriate oncologist at the discretion of the ED physician. Upon providing a referral, their oncologist visits the ED or examines the EMR to determine the patient’s disposition. During off-clinic hour or on holidays, the referral is made to the on-call oncologist. However, even if the patient waits for them, the request for hospitalization may be denied if it is deemed unnecessary by the oncologist.

### National health insurance system in South Korea

In South Korea, health insurance is provided by the National Health Insurance (NHI) system. As compulsory social insurance, major sources of financing are contributions from the insured and government subsidy. All healthcare providers are also obligated to join. The coverage of NHI in 2020 was 97.1% of the population [7].

Insured and dependents undergoing healthcare services must provide a copayment—a share of the total healthcare expenses. Moreover, depending on the insurance provider, inpatients and outpatients are expected to make copayments of 20% and 30%–60%, respectively. These copayments and the insurance premium are covered by the Medical Aid Program for people with low income. For patients with cancer, only 5% copayments are charged for 5 years after the diagnosis for any healthcare services related to cancer treatment. This duration may be extended by an oncologist if certain criteria are met.

Healthcare providers cannot deny treatment to patients with NHI. Patients with NHI receive unrestrained access to their choice of healthcare providers. However, the system recommends starting treatment at a primary care clinic. When a patient wants to receive healthcare services at a tertiary hospital, a patient referral slip, issued by a first- or second-tier hospital, is required. However, emergency medical care is an exception.

### Primary care system in South Korea

Most Organisation for Economic Cooperation and Development (OECD) countries have a well-developed primary care sector, but not South Korea.

Owing to the NHI, patients can access any specialty clinic in community and general hospitals without excessive expenses. Although it has the advantage of being able to receive high quality healthcare services at low cost, this free access increases the frequency of medical consultations. In 2019, South Korea's annual doctor consultation rate was 17.2, which was significantly higher than the OECD average of 6.8 [8]. This unrestrained access also incentivizes patients to choose a tertiary hospital regardless of their disease severity. As a result, the primary care sector is relatively underdeveloped.

### Statistical analysis

IBM SPSS (version 23.0; IBM Corp., Armonk, NY, USA) was used for the statistical analysis. The general characteristics of the study participants were expressed as median and interquartile ranges, and chief complaints were analyzed in terms of frequency and percentage at the time of visit. For continuous variables, the Mann–Whitney test was used, and the Pearson’s chi-squared test was used for categorical variables. The significance level for the test was set at *P* < 0.05.

## Results

### Clinical characteristics

Table 1 shows the clinical characteristics of patients with cancer who visited the ED during the study period. The median age was 66.0 years (range, 57.0–75.0 years); 59.1% of the patients were male, and 40.9% were female. About 92.2% of the total participants accounted for level three and four of the ESI. In the ED and the referral groups, 51.4% and 78.2% accounted for level three ESI, while 41.6% and 12.7% accounted for level four ESI, respectively. There was a significant difference in the ESI between these groups (*P* < 0.001).

**Table 1:** Clinical characteristics of the study patients

Variables	Total (*N* = 607)	ED group (*N* = 387)	Referral group (*N* = 220)	*P*-value
**Age (yr)**	66(57, 75)	67(57, 76)	65.5(57, 73.8)	0.417^a)^
**Sex**				0.163^b)^
Male	359(59.1)	237(61.2)	122(55.5)	
Female	248(40.9)	150(38.8)	98(44.5)	
**Emergency Severity Index**				< 0.001^b)^
Level 1	3(0.5)	2(0.5)	1(0.5)	
Lever 2	36(5.9)	17(4.4)	19(8.6)	
Lever 3	371(61.1)	199(51.4)	172(78.2)	
Lever 4	189(31.1)	161(41.6)	28(12.7)	
Lever 5	8(1.3)	8(2.1)	0(0.0)	
**Vital signs**				
Systolic blood pressure (mmHg)	120(110, 140)	120(110, 140)	120(110, 150)	0.841^a)^
Diastolic blood pressure (mmHg)	80(70, 90)	80(70, 90)	80(70, 90)	0.387^a)^
Body temperature (°C)	36.8(36.4, 37.3)	36.7(36.3, 37.1)	37(36.5, 37.6)	< 0.001^a)^
Heart rate (beats/min)	97(81, 110)	95(80, 110)	99(82, 113)	0.178^a)^
Respiratory rate (breaths/min)	20(19, 21)	20(19, 21)	20(19, 21)	0.180^a)^
**Laboratory findings**				
White blood cell (× 10^3^/μL)	8.3(5.7, 11.4)	8.1(5.5, 10.8)	8.5(5.7, 12)	0.165^a)^
Hemoglobin (g/dL)	11.5(10.4, 12.6)	11.5(10.2, 12.6)	11.7(10.4, 12.7)	0.530^a)^
Platelet count (× 10^3^/μL)	224(163.3, 303.8)	230(167.5, 305.5)	217(158, 303)	0.548^a)^
C-reactive protein (mg/dL)	2.1(0.5, 7)	1.8(0.5, 6)	2.7(0.4, 9.9)	0.036^a)^

Values are presented as median (interquartile range) or number (%)

ESI, level-1 representing the highest acuity and resource utilization and level-5 representing the lowest acuity and resource utilization; Vital signs and laboratory findings were measured upon patient arrival

^a^*P*-value for Mann–Whitney U test. ^b^*P*-value for Pierson chi-square test

Their systolic blood pressure was 120 mmHg (range, 110–140 mmHg), diastolic blood pressure was 80 mm Hg (range, 70–90 mmHg), body temperature was 36.8 °C (range, 36.4 °C–37.3 °C), heart rate was 97 beats/min (range, 81–110 beats/min), and respiratory rate was 20 breaths/min (range, 19–21 breaths/min). In the case of body temperature, the ED group was 36.7 °C (range, 36.3 °C–37.1 °C) and the Referral group was 37.0 °C (range, 36.5 °C–37.6 °C). There was a statistically significant difference in body temperature between the groups (*P* < 0.001); however, it is difficult to establish clinical significance.

A blood test also showed no abnormal findings except that the C-reactive protein level was 2.1 mg/dL (range, 0.5–7 mg/dL), which was slightly higher than the normal range of 0–0.5 mg/dL.

### Chief complaints

Table 2 shows the chief complaints of patients with cancer who visited the ED. Patients with cancer visited the ED with various symptoms; of these, pain was the most common for 40.0% (243 patients), followed by gastrointestinal system symptoms (14.8%, 90 patients), and respiratory system symptoms (11.0%, 67 patients). Followed by fever, weakness, and technical reasons, such as breakdown of medical devices previously mounted on the body or changes in the feeding tube, accounted for 9.9% (60 patients), 7.4% (45 patients), and 7.6% (46 patients) of the patients, respectively. There were no significant differences between the groups except for gastrointestinal symptoms (*P* < 0.001). Specifically, ED physicians cared 24 of 28 patients who complained of nausea and vomiting and 54 of 56 patients who complained of abdominal distension.

**Table 2:** Chief complaints of the patients

Chief complaints	Total (*N* = 607)	ED group N (%)	Referral group N (%)	*P*-value
**Pain**	243(40)	150(38.8)	93(42.3)	0.396
Abdominal pain	142	75	67	
Chest pain	15	11	4	
Back pain	16	16	0	
Extremity	27	13	14	
Pantalgia	43	35	8	
**Fever and chill**	60 (9.9)	31(8.0)	29(13.2)	0.040
**Respiratory**	67 (11.0)	37(9.6)	30(13.6)	0.123
Short of breath	67	37	30	
**Gastrointestinal**	90 (14.8)	80(20.7)	10(4.5)	< 0.001
Gastrointestinal bleeding	6	2	4	
Nausea and vomiting	28	24	4	
Abdominal distension	56	54	2	
**Neurological**	19 (3.1)	9(2.3)	10(4.5)	0.131
Decreased mentality	5	1	4	
Hemiplegia	4	1	3	
Dizziness	6	5	1	
Seizure	2	1	1	
Headache	2	1	1	
**Weakness**	45 (7.4)	24(6.2)	21(9.5)	0.131
**Urinary system**	23 (3.8)	18(4.7)	5(2.3)	0.140
Micturition disorder	19	16	3	
Hematuria	4	2	2	
**For medical procedure**^a^	46 (7.6)	32(8.3)	14(6.4)	0.394
**Others**	14 (2.3)	6(1.6)	8(3.6)	0.100

Values are presented as number (%). *P*-value for Pierson chi-square test

^a^Visits for repair, re-treatment, and various technological procedures due to abnormalities in the existing devices (low-pressure continuous suction unit, feeding tube, Levin tube, percutaneous nephrostomy tube, etc.)

### Pattern of visits

**Fig. 1:**
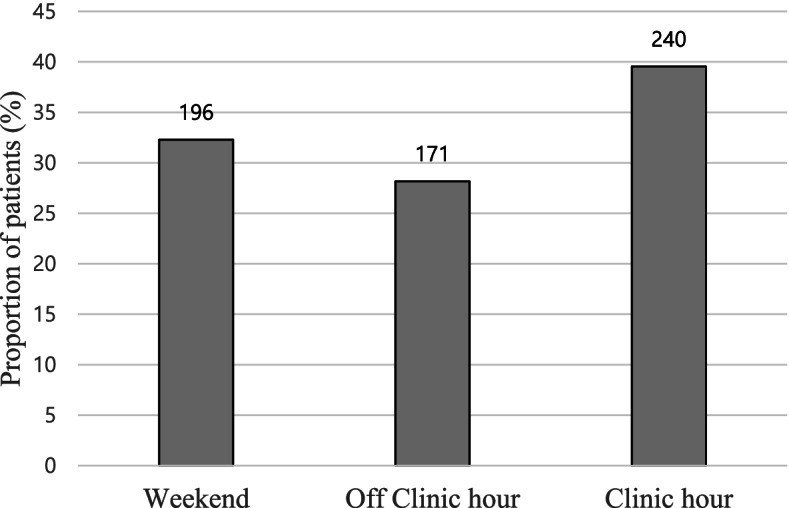
Number of visits by patients with cancer based on clinic hours. Weekend, midnight on Friday to 8 am on Monday; Off-clinic hour, 6 pm to 8 am the following day, Monday through Thursday, and 6 pm to midnight on Friday; Clinic hour, 8 am to 6 pm Monday through Friday. Values are presented as percentage and number

The outpatient clinic was functional from 8 am to 6 pm, Monday through Friday; off-clinic hours were defined as 6 pm to 8 am the following day, Monday through Thursday, and from 6 pm to midnight on Friday, when the outpatient clinic was closed. Weekends were defined as midnight on Friday to 8 am on Monday. There were 240 patients who visited the ED during clinic hours, accounting for 39.5% of the total, 171 patients (28.1%) who visited during off-clinic hours, and 196 patients (32.3%) who visited on weekends and holidays (Fig. 1).

**Fig. 2:**
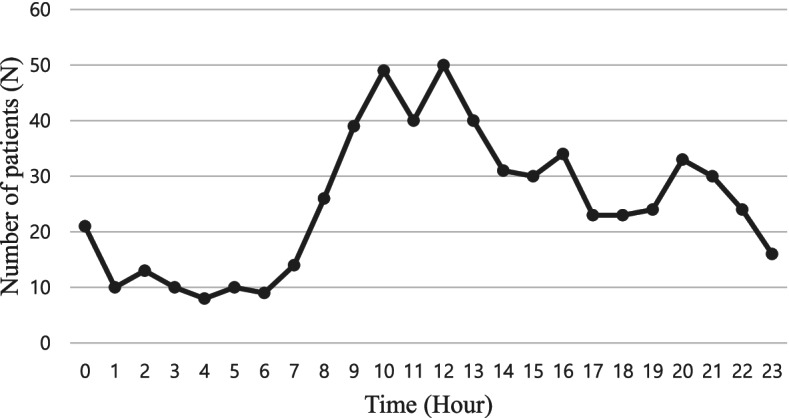
Number of patients with cancer according to time of ED visit

Figure 2 shows the number of patients with cancer according to time of ED visit. The number of patients with cancer who visited the ED at noon was the highest, at 50 patients (8.2%), followed by 49 patients (8.1%) who visited at 10 am and 40 (6.6%) who visited at 11 am and 1 pm. Overall, the number of ED visitors was higher during clinic hours and gradually decreased after 7 pm, with very few patient visits after midnight (Fig. 2).

### Referral to the oncologist and length of ED stay

Of the patients with cancer who visited the ED, 220 patients (36.2%) were referred to their oncologist for hospitalization, and the remaining 387 patients (63.8%) were discharged following symptom improvement after care from an ED physician. Of the 220 referred patients, the actual number who were hospitalized was 170 (77.3%), while 41 (18.6%) patients were denied admission by their oncologist. Six patients (2.7%) were discharged from the ED because the patients decided against hospitalization following referral, and 3 patients (1.4%) died in the ED before hospitalization.

**Fig. 3:**
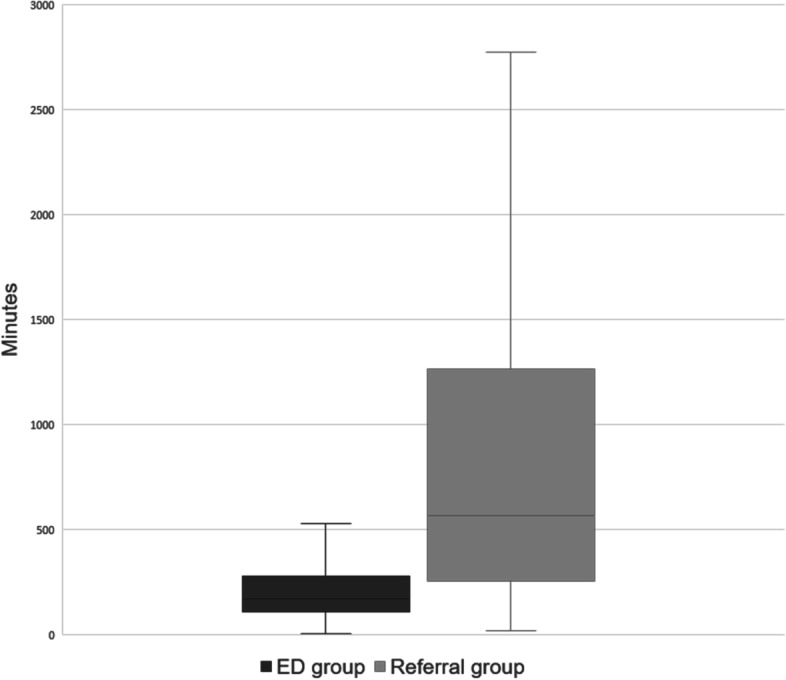
Length of ED stay. ED group: Patients with cancer who presents to the ED and were cared for by the ED physician. Referral group: Patients with cancer who presents to the ED and were referred to their oncologist. Values are presented as median (interquartile range). *P*-value < 0.001

The median length of ED stay was 169 min (range, 110.0–277.5 min) and 566 min (range, 256.5–1263.5 h) in the ED and referral group, respectively, showing a significant difference with a *P*-value < 0.001 (Fig. 3).

## Discussion

In this study, we confirmed that a significant number of patients with cancer visited the ED during a time the outpatient clinic was open, and the most common chief complaint was pain.

Our results demonstrate that 39.5% patients with cancer visited the ED during the daytime on weekdays when the outpatient clinic was open. This means that patients with cancer do not choose to go to the ED as an alternative only on weekends and nights when their oncologist is off duty. This is a very interesting result considering the characteristics of chronically ill patients including those with cancer: most chronically ill patients are highly dependent on their specialty physicians depending on their chronic conditions. The reason for this could be the fact that it is difficult to see their oncologist without an appointment despite sudden symptom presentation necessitating medical care.

Several studies have revealed that pain is the most common reason for sudden medical care visit to the ED [3, 9–12], which is consistent with current study results. Considering the nature of pain, it is impossible to predict its occurrence, but patients may need medical care urgently. However, it is typically not easy to obtain a same-day appointment at the outpatient clinic or visit without an appointment due to advance bookings from other patients for that day. This often motivates patients with cancer to visit the ED. The ED is open throughout the day, and the doctors there provide treatment at any time, without the need to book an appointment [13, 14]. The absence of suitable alternatives other than visiting the ED, as well as the anxiety of patients with cancer about the disease and their psychological desire to seek medical treatment quickly, can be additional factors leading them to visit the ED [4–6, 15]. While the oncology infusion clinics or acute care clinics can be a good alternative [16], there are difficulties in countries like South Korea, where these clinics are not active.

The difficulty of booking an outpatient appointment with their oncologist at a tertiary hospital is associated with the consultation rate of patients. In South Korea, the medical consultation rate is thrice that of the average of OECD countries due to the low medical burden. In addition, owing to the NHI coverage, 61.8% of the patients with cancer flock to tertiary hospitals, which account for only 0.06% of all medical institutions. Thus, making an appointment is bound to be even more difficult [17]. On the other hand, many patients with cancer visit the ED of tertiary hospitals when they suddenly develop symptoms, as it provides high-quality care at any time. Particularly, patients usually come to the ED, operated by the tertiary hospital in which their oncologists work. This is to facilitate easier access to medical records in the same hospital, so that even if they receive medical care from a new ED physician, they can expect continuity in their existing treatment plan, and it is also convenient to request their oncologist for hospitalized.

The problem is that after completion of medical care, a patient wishes to be hospitalized to their oncologist even though it is not medically necessary and requests a referral. Upon comparing the ESI between the ED and referral groups, the ED group patients mostly reflected levels three and four, while the referral group patients reflected level three the most. However, since most patients in level four and five were cared for in the ED group, the population of the ED group increased statistically, but the absolute number of level one through three patients cared for was similar in both groups. A clear causal relationship could not be established between the patient severity and the referral, as the referral also involved subjective factors such as the patient's earnest request. Indeed, 18.6% of referred patients were eventually denied admission by their oncologist. Owing to the NHI System, characterized by low copayment and wide coverage, several patients who can be discharged after completion of medical care in the ED, request a consultation with their oncologist and hospitalization.

Patient fear may be another reason that patients with cancer stay in the ED for a long time. The fear that pain might be a symptom of cancer-related deterioration, and the fear of getting sick again after returning home makes patients hesitant to leave the ED and want to be hospitalized even if the medical evidence is weak [4–6, 15].

Whatever the reason, collaborative care through referral can contribute to improving the quality of care. However, it is inevitably time-consuming. For this reason, referral often acts as a weakness in care that requires prompt action, such as pain treatment. This study revealed that patients stayed in the ED for 566 min when referred to the oncologist, while it took 169 min for them to receive care from an ED physician. It indicates that referral to the oncologist required approximately 397 min longer length of stay in the ED (Fig. 3). Several factors, such as patient severity, are mixed in the consumption of ED stay duration for patients referred to the oncologist. Undeniably, the inability of the oncologist to quickly arrive at the ED is also a factor. Most oncologists work in their own wards and schedule outpatient care rather than resident care in the ED. When a patient in the ED is referred, it is not possible to leave the patient who was being treated at that moment; thus, it takes time to visit the ED after completing the treatment. At least several hours after referral are spent waiting for the oncologist, without any change in the initial treatment plan. Although this is an unavoidable aspect, from the patient's point of view, this waiting is not beneficial.

In case of oncologic emergencies such as pain, treatment in the ED is recommended rather than waiting at an outpatient clinic. And, do not make the patients wait for the oncologist for merely inpatient admission counseling even after the completion of treatment, as it only causes extended length of ED stay and ED overcrowding.

Rather than responding to a patient's request for referral and admission with weak medical evidence, it seems more advantageous for patients receiving ED physician’s faster medical care and detailed explanation of the patient's current cancer-related condition based on their test results. It will help the patients with cancer get rid of their fear, reduce unnecessary hospitalization requests and time spent in the ED.

## Conclusions

Pain was the most common reason for visiting the ED among patients with cancer. Collaborative care through referral to the oncologist has considerable limitations in terms of timeliness. This disadvantage is more prominent in handling situations that are emergent and difficult to bear, such as pain. Therefore, it would be more beneficial for patients with cancer visiting the ED to be quickly discharged from the ED physician’s active care for their symptoms. This usage of ED services will reduce unnecessary waiting time.

## Data Availability

The datasets generated and analyzed during the current study are not publicly available due to the protection of participant’s identities, but are available from the corresponding author on reasonable request.

## Abbreviations

ED: Emergency department
EMR: Electronic medical records
ICD: International classification of diseases
ESI: Emergency severity index
NHI: National health insurance
OECD: Organisation for economic cooperation and development
